# A data package for abstractive opinion summarization, title generation, and rating-based sentiment prediction for airline reviews

**DOI:** 10.1016/j.dib.2023.109535

**Published:** 2023-09-01

**Authors:** Ayesha Ayub Syed, Ford Lumban Gaol, Alfred Boediman, Tokuro Matsuo, Widodo Budiharto

**Affiliations:** a Department of Doctor of Computer Science – BINUS Graduate Program, Bina Nusantara University, Jakarta, Indonesia; b Department of Econometrics and Statistics - The University of Chicago, Booth School of Business, USA; c Graduate School of Industrial Technology, Advanced Institute of Industrial Technology, Tokyo 140-0011, Japan; d Department of M-Commerce and Multimedia Applications, Asia University, Taichung 41354, Taiwan; e Computer Science Department, School of Computer Science, Bina Nusantara University, Jakarta 11480, Indonesia

**Keywords:** Pretrained language models, Abstractive summarization, Domain adaptation, Sentiment classification, Customer reviews

## Abstract

Customer reviews are valuable resources containing customer opinions and sentiments toward the product. The reviews are informative but can be quite lengthy or may contain repetitive information calling for opinion summarization systems that retain only the significant opinion information from the review. Abstractive summarization is a form of text summarization that generates a summary mimicking a human-written summary [1]. When pretrained language models are finetuned for abstractive review summarization, there usually occurs a problem known as the ‘domain shift’, because the source and target domains exhibit data from varying distributions [2]. This issue results in performance degradation of the model at the target end. This paper contributes a data package comprising of an annotated abstractive summarization dataset (annotated_abs_summ) of airline reviews having 500 reviews and abstractive summary pairs, a dataset (review_titles_data) consisting of 7079 reviews and review title pairs for review title generatioon or domain adaptive training [3] to address the domain shift problem for abstractive opinion summarization and, an annotated reviews dataset (annotated_sentiment) for rating-based sentiment classification. All datasets have been collected from the Skytrax Review Portal via web scraping using Python programming language. The datasets have several potential use cases. The abstractive summarization dataset can serve as a benchmark dataset for airline review summarization. The dataset for domain adaptive training can be used as a standalone dataset for review title generation. The dataset for sentiment analysis is multipurpose having columns like user rating and recommendation value, that can be used for statistical analysis like finding correlation between these data items as well as for other Natural Language Processing (NLP) tasks like predicting rating or recommendation value from the customer reviews. The datasets can be extended using various data augmentation techniques [4,5]. Moreover, the datasets are related and can be collectively used to develop a multi-task learning model [6] for better learning efficiency and improved performance.

Specifications Table

**Table:** 

Subject	Artificial Intelligence, Data Science
Specific subject area	Natural Language Processing: Abstractive Summarization and Sentiment Analysis of Airline Reviews
Type of data	Text, Table
How the data were acquired	The airline reviews data has been collected from the Air travel review website https://www.airlinequality.com/ via web scraping using Python programming language and Google Colaboratory platform.
Data format	Raw Labeled Analyzed
Description of data collection	The primary data was collected by specifying the URL of various airline review webpages and finding the elements of interest such as review, title, rating, recommendation value, etc. The scrapping process was facilitated using Python ‘requests’ and ‘beautiful soup’ library packages. The collected datasets were checked for duplicates and missing values. The reviews with missing titles or other elements were removed from the datasets. For the annotated_abs_summ dataset, the reviews were scraped from the website and the abstractive summaries were manually written. For review_titles_data, reviews and review titles were scrapped directly from the website. For the annotated_sentiment dataset, reviews, review titles, ratings, and recommendation values were scraped from the website while the sentiment value is assigned based on customer rating value. The data comprises reviews from multiple airlines mixed to create a diverse dataset for Natural Language Processing tasks.
Data source location	Skytrax https://www.airlinequality.com/
Data accessibility	Repository name: Mendeley Data Data identification number: 10.17632/pc6fxc95h5.1 Direct URL to data: https://data.mendeley.com/datasets/pc6fxc95h5/1

## 1 Value of the Data

• The data is particularly useful for training deep transfer learning models for title generation, abstractive summarization [[1], [5]], and sentiment analysis of airline reviews.
• The title generation data is applicable for domain adaptive training [[2], [3]] of pretrained language models to improve performance on the target language generation tasks for the airline reviews domain.
• The data can be beneficial for Natural Language Processing research community, airline business research companies, or other interested parties.
• The airline reviews contain sentiments about various product features expressed in a single review. In addition to an overall sentiment prediction task, the data can serve as a good dataset for fine-grained aspect-based sentiment classification.
• The data can be collectively utilized to develop a hybrid summarization and sentiment analysis framework or a multitask deep learning model [6] for better target task learning and further gains in performance improvement. Further, the datasets can be extended using the data augmentation techniques [4].

## 2 Objective

The airline reviews are generally different from other existing review datasets in a manner that these reviews are generally lengthy, have opinions expressed on multiple features, and contain mixed sentiments, all in a single review. These characteristics make the airline review dataset challenging for certain NLP tasks like summarization and sentiment classification. The reasoning behind the generation of this data package is to train and develop better deep-learning models for abstractive summarization and sentiment classification that can cope effectively with such challenging datasets.

We compared the airline review dataset with the Yelp review dataset in terms of some basic statistics to highlight the lengthiness of airline reviews. These statistics are shown in Table 1. The average character count measures the average of character counts across all reviews in the dataset, the max character count measures the maximum length (in characters) of a review in the dataset, the min character count measures the smallest length (in characters) of a review in the dataset, the average word count measures the average number of words across all reviews in the dataset, the maximum and minimum word counts measure the number of maximum and minimum words in reviews across the datasets, and the mean word length takes the average of mean word length across all the reviews in the datasets. It can be noted that almost all statistics indicate the lengthy nature of airline reviews.

**Table 1:** Statistics of airline reviews vs Yelp restaurant reviews.

Statistics	Airline Reviews	Yelp Restaurant Reviews
Average character count	825.28	489.83
Max character count	4908	4961
Min character count	111	19
Average word count	149.79	90.44
Max word count	957	949
Min word count	17	3
Mean word length	4.56	4.49

**Fig. 1:**
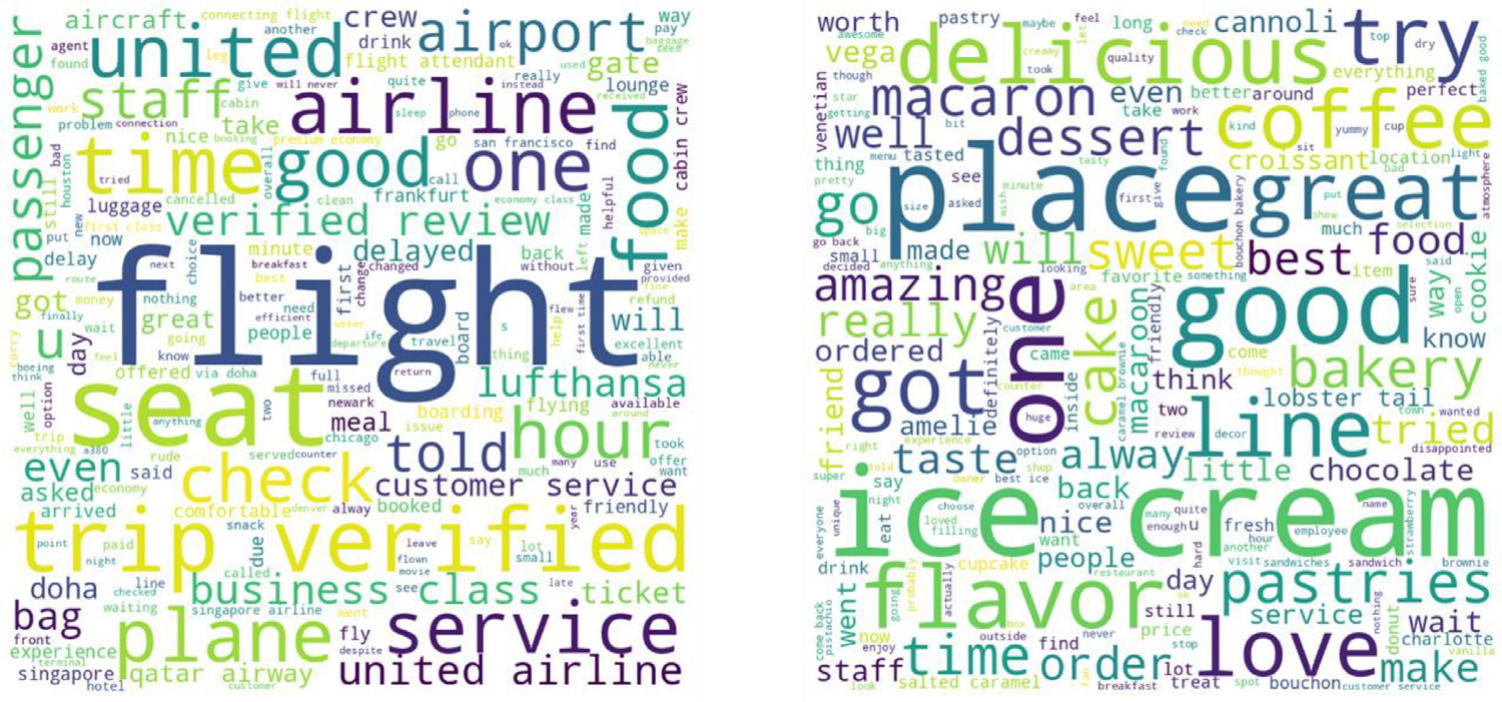
Word clouds generated from airline reviews and Yelp restaurant review datasets.

Fig. 1 adds an explanation to the aspect-rich nature of airline reviews. From the word cloud of the airline review dataset, the most frequent words or topics are observed as ‘flight’, ‘seat’, ‘service’, ‘plane’, ‘time’, ’airline’, ‘food’, ‘staff, ‘airport’ etc. For the Yelp reviews dataset, most of the high-frequency words or topics are related to ‘place’, kinds of food like ‘ice cream’, ‘coffee’, and ‘pastries’, and sentiments related to place and food. The number of aspects discussed in airline reviews is certainly more as compared to restaurant reviews, thus requiring advanced methods for language processing (summarization and sentiment classification) tasks.

## 3 Data Description

The data repository has three data files named review_titles_data, annotated_abs_summ, and annotated_sentiment.

## 4 The Review_Titles_Data Dataset

The review_titles_data has 7079 rows and 2 columns. The columns are named ‘review’ and ‘title’. The ‘review’ column contains reviews written by airline customers and the ‘title’ column contains the titles for corresponding reviews. A sample from the dataset is presented in Table 2.

**Table 2:** A sample from the review_titles_data dataset.

Review	title
? Verified Review | Vietnam Airlines seemed to be a reliable airline with good service on board. Should regretfully admit that this was not so. I know that our flight was based in Nha Trang, but the plane was not clean, as if it was a return flight. On the luggage racks lay garbage, the portholes were dirty, in the pocket of the seats was also garbage. There was also a lack of on-board service on domestic flights - only water is offered. As you can see, the onboard magazine is unpleasant to hold. I can not recommend, I will avoid the airline, including on long-haul flights.	“the plane was not clean”

**Fig. 2:**
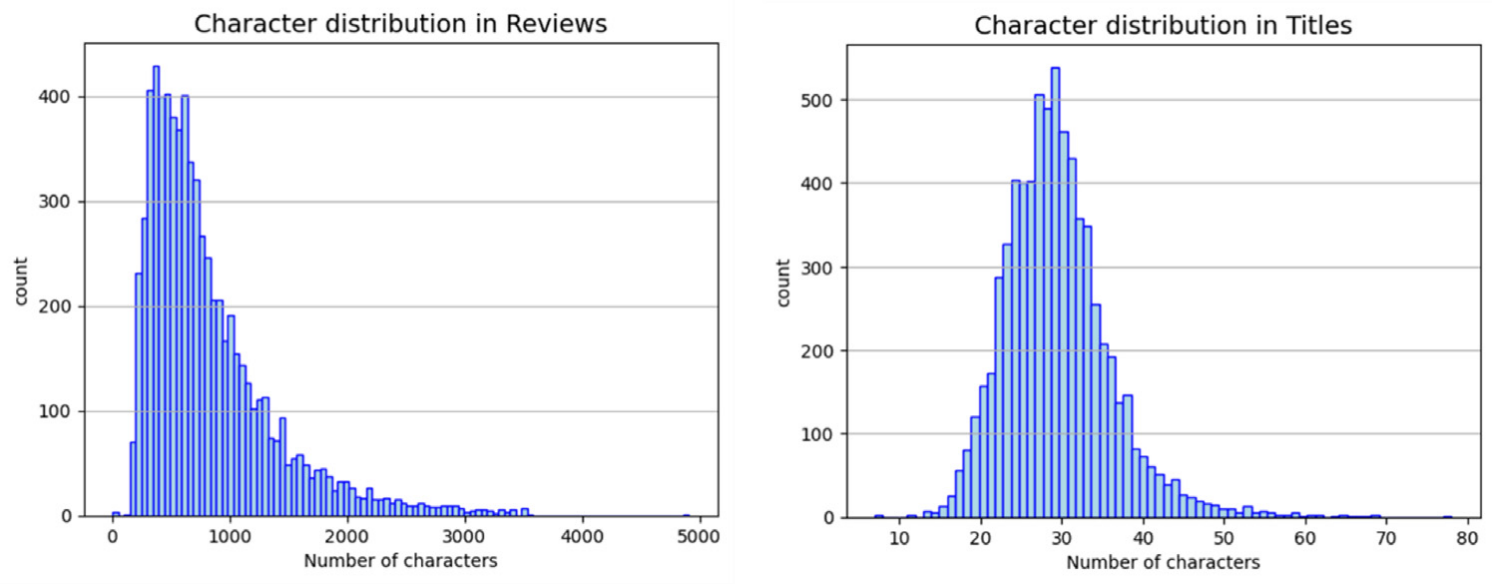
Character distribution across reviews and titles in the review_titles_data dataset.

Fig. 2 shows the character distribution in the review_titles_data dataset. The left-hand side indicates the character distribution across reviews and the right-hand side gives the distribution across titles. From Fig. 1, most reviews lie in the range of 200 to 1200 characters and most titles have a character distribution between 20 and 40 characters.

**Fig. 3:**
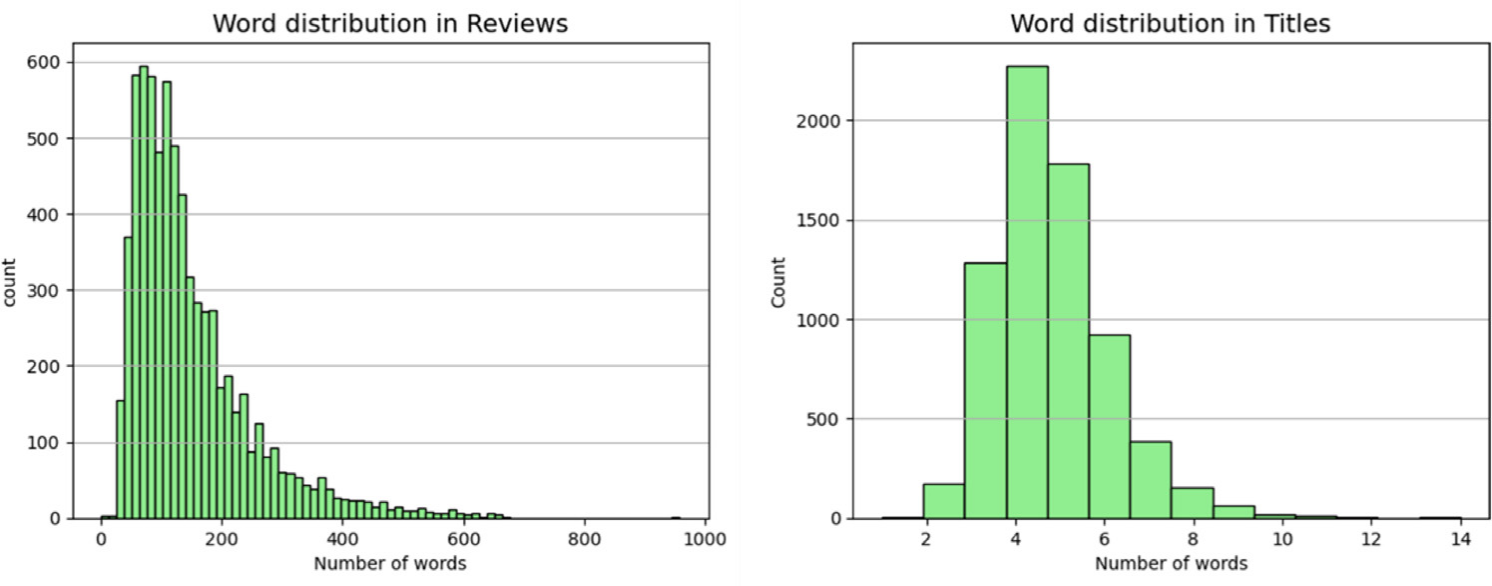
Word distribution across reviews and titles in the review_titles_data dataset.

Fig. 3 illustrates word distribution across reviews and titles in the review_titles_data dataset. The number of words in most of the reviews is between 50 and 300 while most titles have a word range between 3 and 7 words.

## 5 The Annotated_abs_summ Dataset

The annotated_abs_summ dataset has 2 columns and 500 rows. The columns are named ‘review’ and ‘summary’. The first column ‘review’ contains the review text posted by an airline customer. The second column ‘summary’ is the manually written abstractive summary of the corresponding review. A sample entry from the dataset is shown in Table 3.

**Table 3:** A sample from the annotated_abs_summ dataset.

Review	summary
Hong Kong to Brisbane via Taipei. Hong Kong to Taipei was Ok, service was reasonable. Taipei to Brisbane was uncomfortable and food selection was poor. While in Australia China Airlines canceled the return leg Taipei to Hong Kong. I made 2 phone calls and 1 email to their Taipei office explaining that I still wanted to continue with the Brisbane to Taipei leg. Before the flight I received and email stating the refund would be HK$ 4055. At Brisbane I was advised by China Airlines staff to make my own ongoing arrangements. After there flight I received another email saying the refund was now HK$ 460, which is 3.7% of the ticket cost. Really bad customer service at Brisbane and Taoyuan airport and subsequently through emails.	Service for Hong Kong to Taipei was satisfactory while for Taipei to Brisbane, it was not good. There were no good choices for food. Later, due to flight cancelation, there were problems with the refund. The customer service at Brisbane and Taoyuan airports was substandard.

**Fig. 4:**
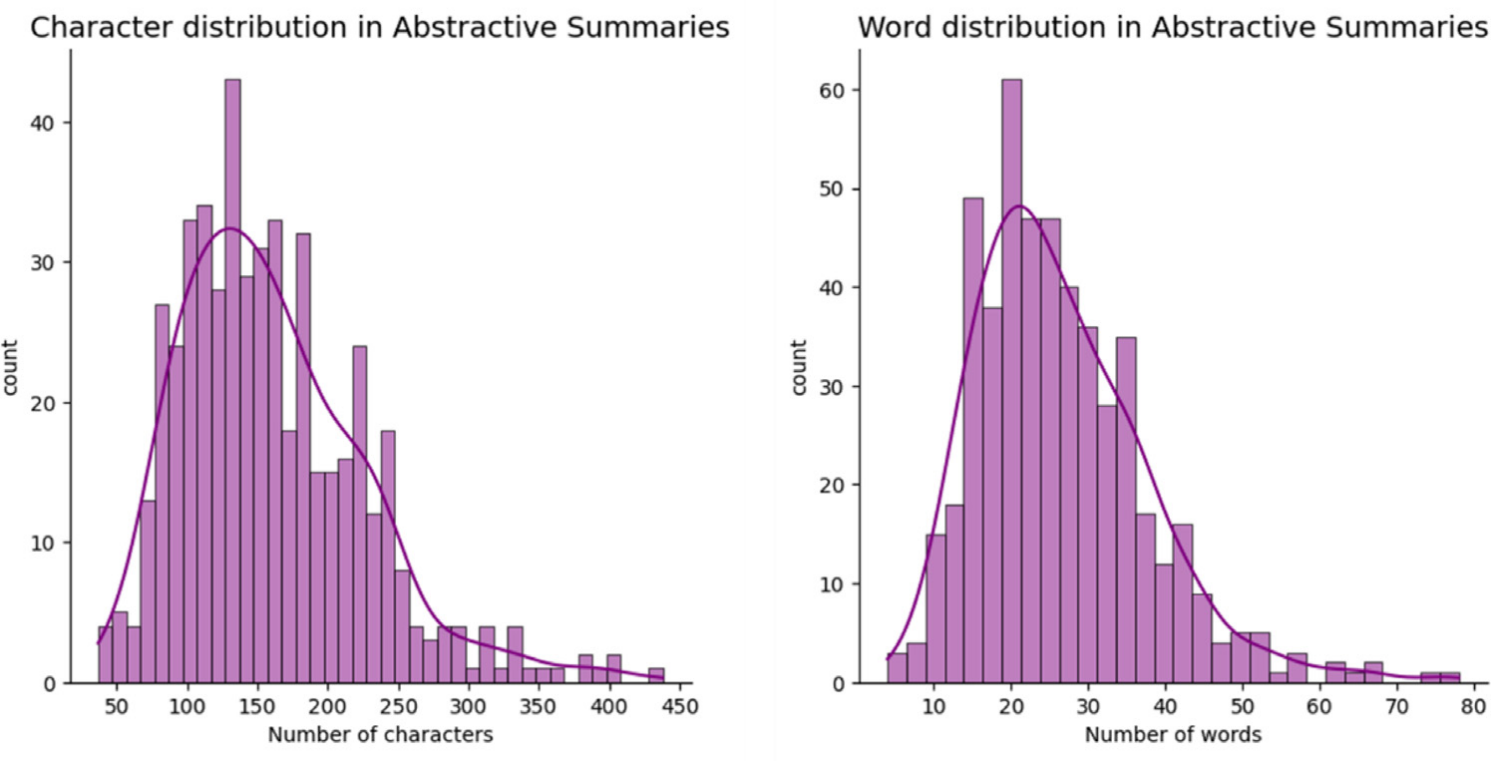
Character distribution and word distribution across abstractive summaries in the annotated_abs_summ dataset.

**Fig. 5:**
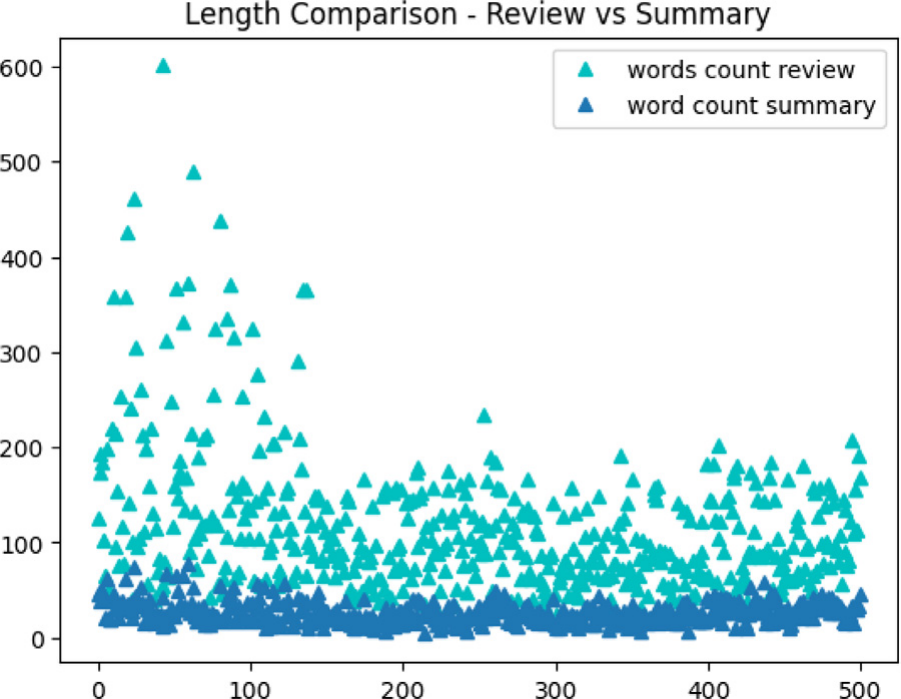
Review length vs summary length (in words).

Fig. 4 describes the data distribution in the annotated_abs_summ dataset. Most of the summaries have a character distribution between 100 and 250 characters while the majority of summaries have a word distribution between 15 and 40 words. Fig. 5 reflects the difference between the review length and summary length in the dataset. Table 4 reveals mean values for review length, summary length, and the percentage of compression ratio between the review and summary in the annotated_abs_summ dataset.

**Table 4:** Statistics - annotated_abs_summ dataset.

Number of Reviews in annotated_abs_summ dataset	Mean Review Length (in words)	Mean Summary Length (in words)	Mean Compression Ratio (%)
500	115.742000	26.382000	26.3

## 6 The Annotated_Sentiment Dataset

**Fig. 6:**
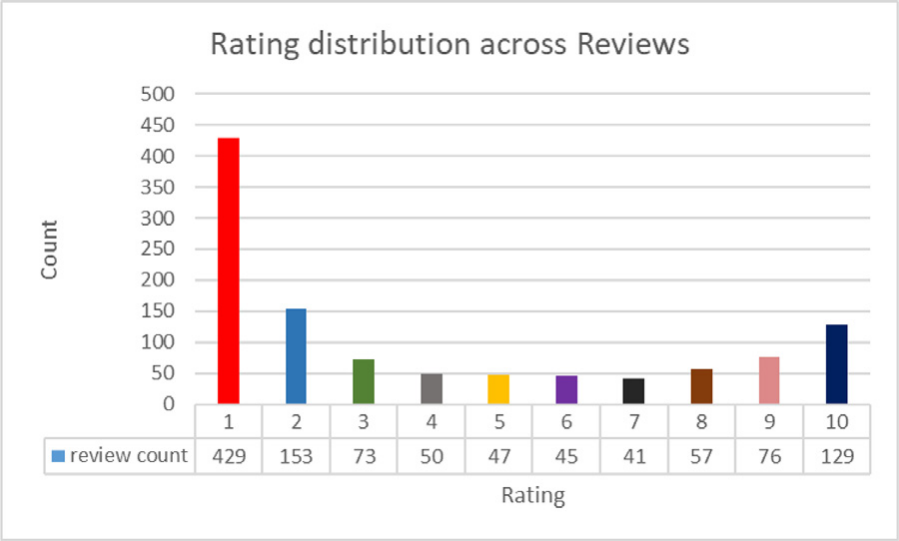
Customer ratings across reviews in the annotated_sentiment dataset.

**Fig. 7:**
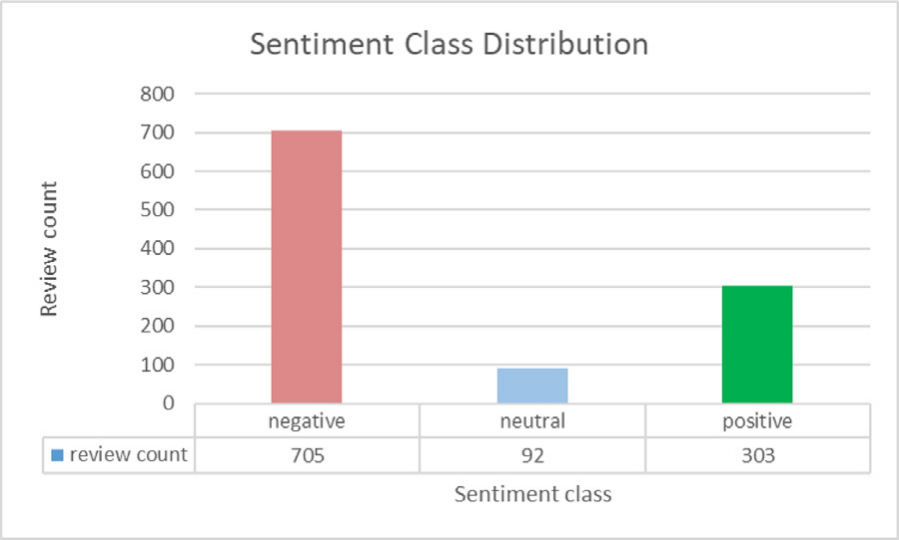
Sentiment class distribution across reviews in the annotated_sentiment dataset.

**Fig. 8:**
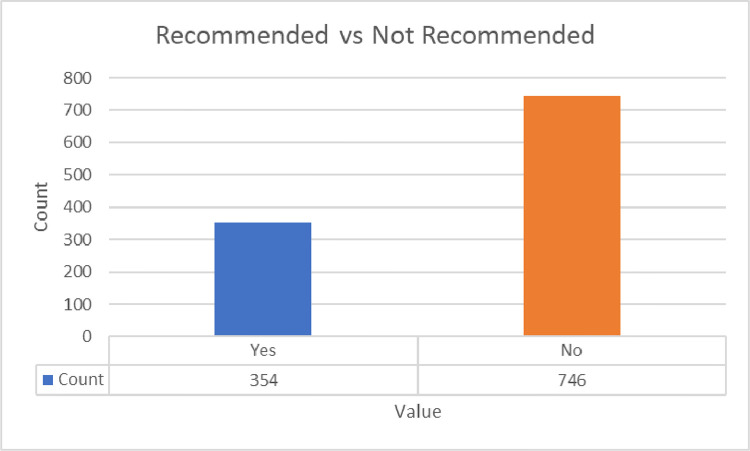
Customer recommendation across reviews in the annotated_sentiment dataset.

The annotated_sentiment dataset has 1100 rows and 5 columns named ‘review’, ‘title’, ‘rating’, ‘recommended’, and ‘sentiment’. The ‘review’ column contains the review posted by an airline customer. The ‘title’ column has the title of the corresponding review. The ‘rating’ column shows the overall rating given by the airline customer. The rating values range between 1 and 10, where 1 is the worst and 10 is the best. The next column ‘recommended’ indicates whether the airline is recommended by the reviewer or not. It contains two values ‘yes’ and ‘no’. The last column named ‘sentiment’ is annotated based on the rating value. The values range from 0 to 2, where 0 represents negative sentiment, 1 denotes neutral sentiment, and 2 stands for positive sentiment. The rating, sentiment, and recommendation distributions across the dataset are presented in Fig. 6, Fig. 7, and Fig. 8. A sample data entry from the dataset is exhibited in Table 5.

**Table 5:** A sample from the annotated_sentiment dataset.

Review	Title	rating	Recommended	Sentiment
Trip Verified | Tried booking 3 tickets on Air Canada website which said an error has occurred. My card was charged but I did not receive a valid ticket number. The reservation temporarily appeared but also later disappeared off my account. No response from Air Canada - where is my tickets or my money? I tried calling and emailing Air Canada with no response.	“No response from Air Canada”	2	No	0

## 7 Experimental Design, Materials and Methods

### 7.1 Data collection

**Fig. 9:**
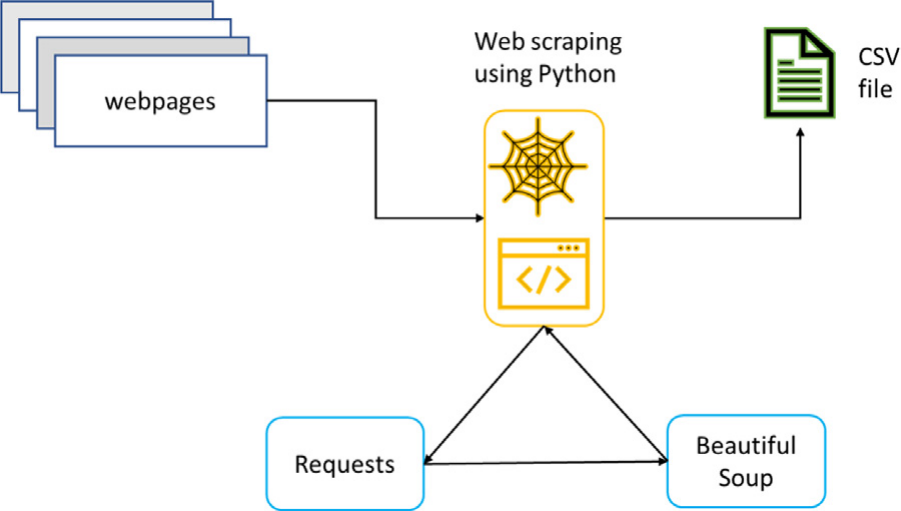
The methodology for the data collection process.

The datasets have been collected from the Skytrax review portal (www.airlinequality.com) via web scraping using Python programming language. In this experiment, web scraping was supported using Python Requests and BeautifulSoup libraries. The basic methodology for web scraping used in this experiment is illustrated in Fig. 9. After importing the necessary libraries, the Requests library was used to put forward a request to the webpage by specifying the URL. The Beautiful Soup package was utilized to obtain the data from the requested web page. The desired HTML tags were located depending on the dataset requirements and finally eliminated to get the data in plain text. For review_titles_data and annotated_abstractive_summ dataset, the HTML tags for review and title were located and retrieved. For the annotated_sentiment dataset, HTML tags for review, title, rating, and recommendation were added. The classes and attributes were also added where required.

## 8 Data Annotation

The data collection phase was followed by the data annotation phase. For the annotated_abs_summ dataset, two language experts were involved in writing the review summaries. The annotation process took 3 to 4 months. The summary for each review was written based on the following criteria:

1. An overall opinion of the customer towards the airline.
2. The customer's expression of opinion towards the mentioned aspects e.g., food, seating, check-in, crew service, etc.
3. An emphasis on language structure and grammar.
4. A special consideration towards the inclusion of some novel words in the summary.
5. Keeping the size of the summary around 30–40% of the original review.

The quality of the summaries is maintained during the writing process as well as assessed after the annotation completion. The summary quality assessment was based on measures such as fluency, coherence, non-redundancy, informativeness, and sentiment. These measures were adopted from [7] and are defined in Table 6.

**Table 6:** Definition of summary quality measures.

Quality Measure	Definition
Fluency	Readable, grammatically correct, understandable
Coherence	Well-structured and organized information
Non-redundancy	No repititive information
Informativeness	Good information coverage
Sentiment	Conveys customer sentiment

For the annotated_sentiment dataset, the sentiment class annotation was derived from customer rating. The annotation guidelines are listed in Table 7.

**Table 7:** A rating scale for sentiment class annotation.

Rating	Sentiment Class
1–4	Negative (0)
5–6	Neutral (1)
7–10	Positive (2)

## 9 Data Analysis

**Fig. 10:**
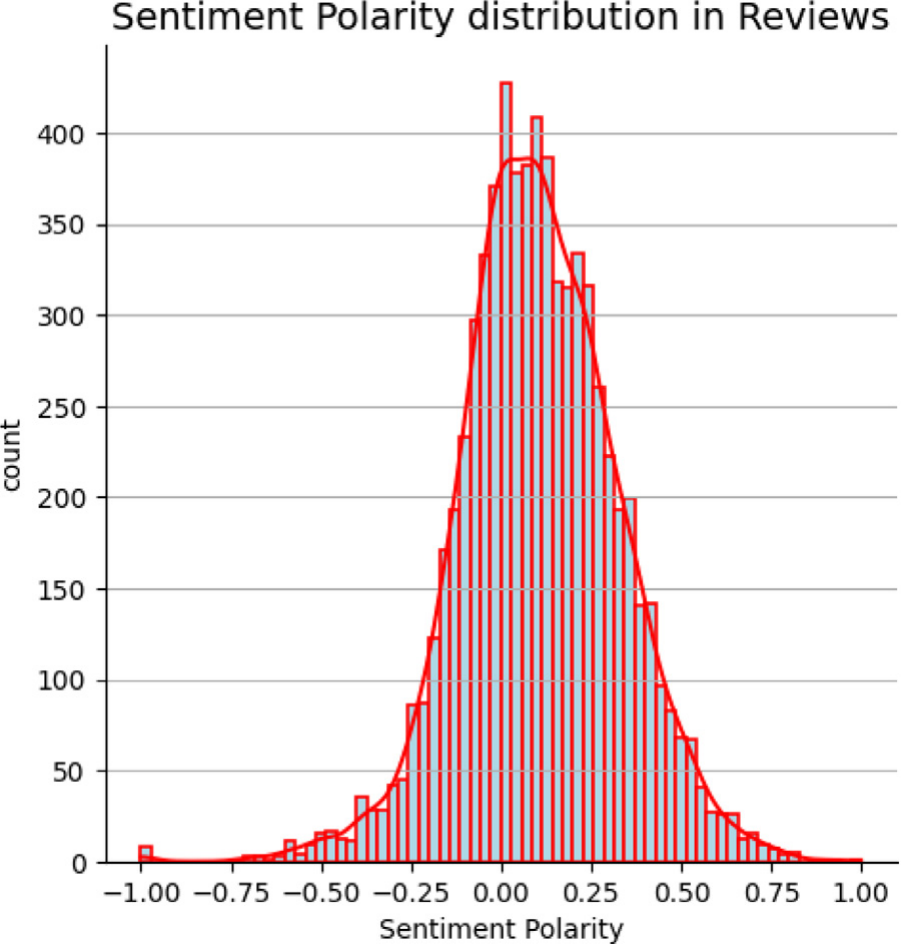
Sentiment polarity distribution across reviews in the review_titles_data dataset.

The review_titles_data was analyzed for sentiment polarity using the Python textblob package. The resulting distribution is presented in Fig. 10. The sentiment polarity values range between −1 and +1, where negative values indicate a negative sentiment, zero indicates a neutral sentiment and positive values denote positive sentiments. The results show an almost similar distribution of positive and negative sentiments across reviews in the dataset.

**Fig. 11:**
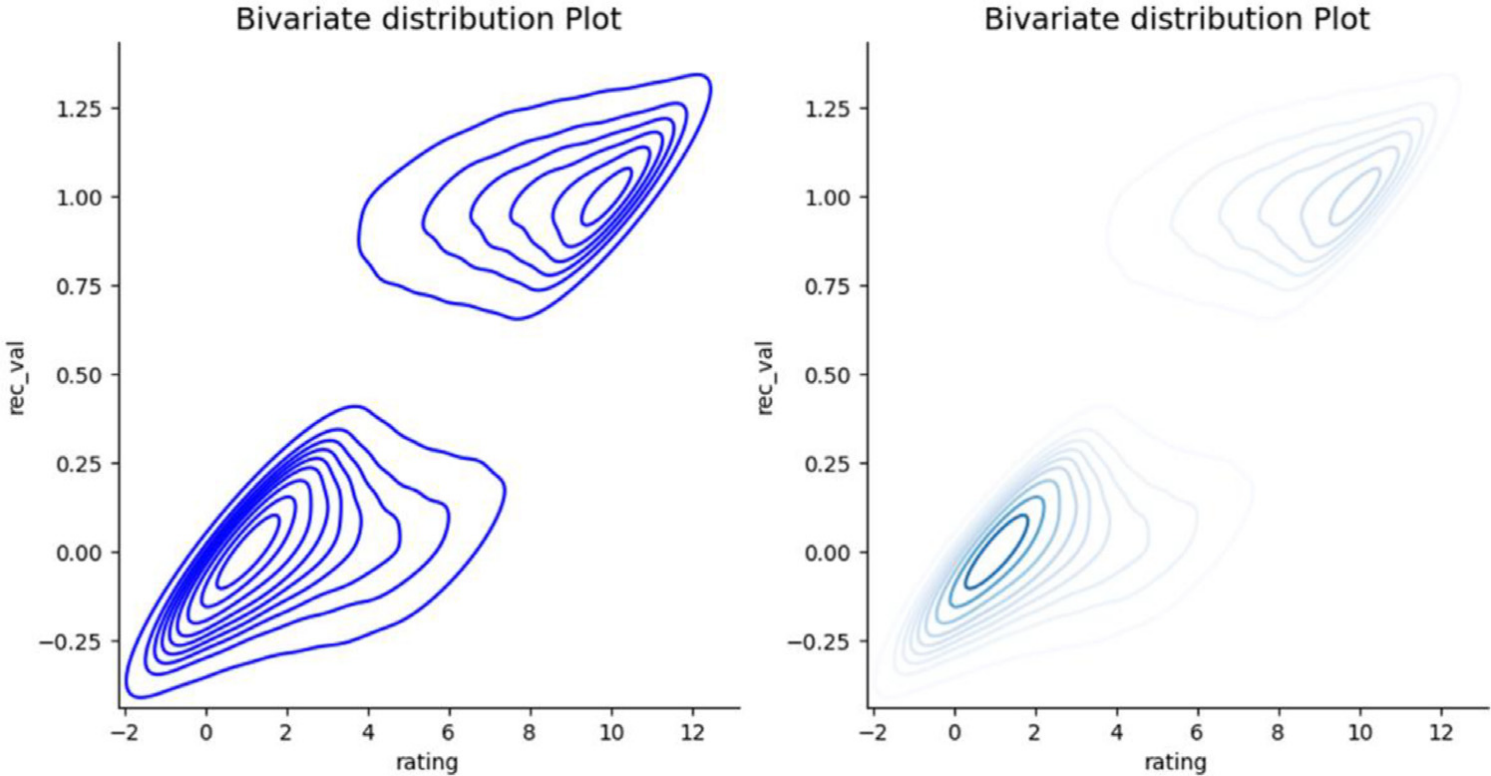
The joint probability distribution plot of customer rating and recommendation value.

Fig. 11 illustrates the bivariate probability distribution with customer rating and recommendation value from the annotated_sentiment dataset. The joint distribution of rating and rec_val is plotted as Kernel Density Estimate (KDE) using a contour plot. The regions with higher density are seen in dark colors on the right-hand side of Fig. 11. The graph shows a positive correlation between rating and recommendation value, i.e., higher rating scales are associated with positive recommendation values while lower rating scales are linked to negative recommendation values.

**Fig. 12:**
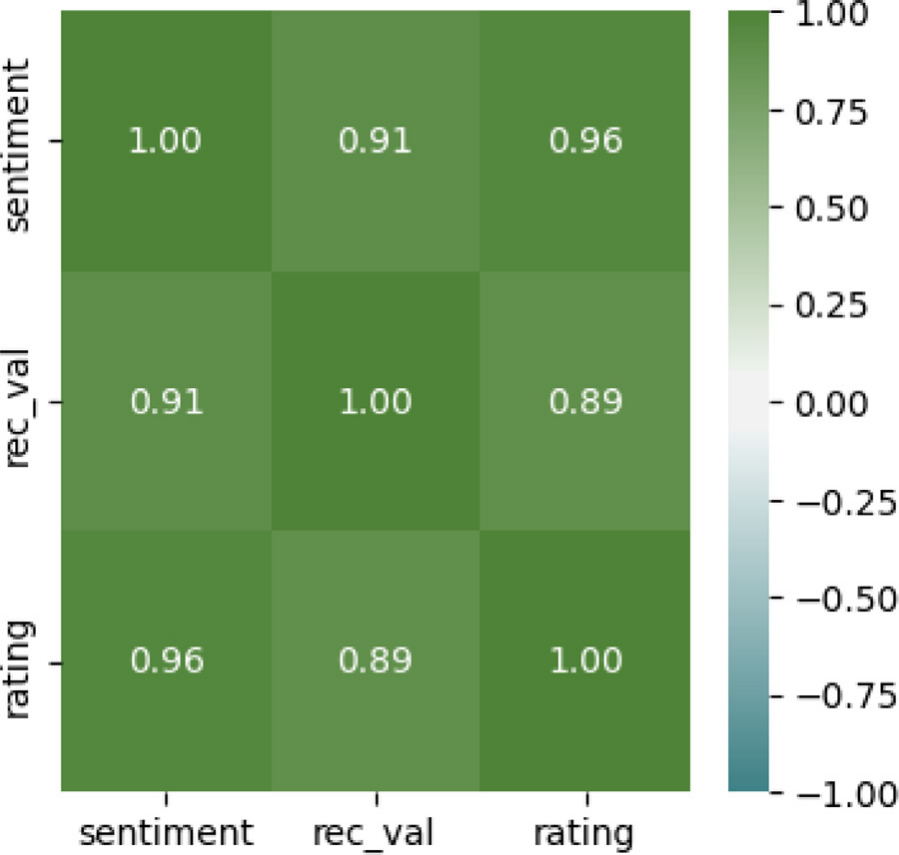
The correlation heatmap – customer rating, recommendation, and sentiment.

Fig. 12 shows a correlation heatmap that represents correlation patterns between customer rating, recommendation value, and sentiment. From the correlation matrix, rating, and sentiment, having a correlation value of 0.96 are the most closely related. The correlation between recommendation value and sentiment is 0.91 while between rating and recommendation value, the correlation is 0.89. All the variables show a close association with one another.

## 10 Discussion

There are some existing airline review datasets crawled from the Skytrax review portal. The major differences between the existing datasets and our data package are:

1. Most of the existing datasets are outdated, ours is current and up to date.
2. Most of the existing datasets have been used for statistical and comparative analysis e.g., to determine the best airline or airport, etc. Still, there are some existing works related to the Natural Language Processing (NLP) analysis of airline reviews, for example, the work of [8]. In such works, NLP algorithms were applied directly to get insights from the data, and the researchers did not utilize or contribute labeled datasets. Our data package is intended for NLP research of airline reviews specifically for supervised training of machine learning and deep learning models for the tasks of abstractive summarization, title generation, and sentiment classification of airline reviews.
3. The existing datasets usually contain only data scraped from the website. In the case of our datasets, we added annotated/labeled data columns specifically for abstractive text summarization and rating-based sentiment classification.

## Ethics Statements

The data is collected from the airline reviews portal merely for research purposes and the participant data has been fully anonymized.

## CRediT Author Statement

**Ayesha Ayub Syed:** Conceptualization, Methodology, Data Curation, Formal Analysis, Writing; **Ford Lumban Gaol:** Conceptualization, Review & Editing, Resources; **Alfred Boediman:** Investigation, Validation; **Tukoro Matsuo:** Resources; **Widodo Budiharto:** Supervision.

## Declaration of Competing Interest

The authors declare that they have no known competing financial interests or personal relationships that could have appeared to influence the work reported in this paper.

## Data Availability

Airline reviews dataset for Abstractive Summarization and Sentiment Classification (Original data) (Mendeley Data).
